# COVID-19-Related Scientific Literature Exploration: Short Survey and Comparative Study

**DOI:** 10.3390/biology11081221

**Published:** 2022-08-16

**Authors:** Bahaj Adil, Safae Lhazmir, Mounir Ghogho, Houda Benbrahim

**Affiliations:** 1 TicLAB, International University of Rabat, Sala el Jadida 11103, Morocco; 2 Faculty of Engineering, University of Leeds, Leeds LS2 9JT, UK; 3 ENSIAS, Mohamed V University, Rabat 11000, Morocco

**Keywords:** COVID-19, exploratory search, machine learning, document retrieval

## Abstract

**Simple Summary:**

The COVID-19-related literature has known a surge since the beginning of the pandemic. This surge prompted the creation of multiple literature exploration systems to help automate the exploration of scientific articles. In this work, we survey multiple COVID-19 literature exploration systems by exploring their most discriminative characteristics, give general design principles for these systems, and describe some of their limitations.

**Abstract:**

The urgency of the COVID-19 pandemic caused a surge in the related scientific literature. This surge made the manual exploration of scientific articles time-consuming and inefficient. Therefore, a range of exploratory search applications have been created to facilitate access to the available literature. In this survey, we give a short description of certain efforts in this direction and explore the different approaches that they used.

## 1. Introduction

Due to the vast expansion of the COVID-19 literature (according to LitCOVID [1] website, more than 258,000 unique papers had been published on PubMed before 10 July 2022), there was a need to create information management and retrieval systems for the COVID-19 literature. The data science community responded to this urgent need by creating and deploying dozens of applications to provide researchers with easy access to the COVID-19 literature. These applications mainly focus on text mining [2] and its related tasks (e.g., document retrieval [3], question answering [4], passage retrieval [5], summarization [6], etc.) in order to organize and access relevant knowledge effortlessly. Several public competitions and common tasks, such as the CORD-19 and TREC-COVID initiatives [7,8], further encouraged such efforts.

**Figure 1:**
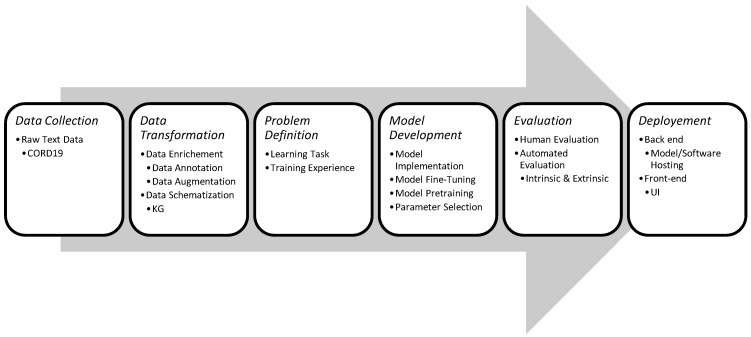
Application Development Phases.

In this work, we explore the COVID-19 literature exploration applications, which we can classify as one of two categories relative to the format of the search results; (a) textual search engines, and (b) visual search engines. The first category comprises query-oriented applications that extract information from the COVID-19 literature using queries. The second class of applications is used mainly for the bibliometric study of the COVID-19 literature coupled with visual interactive or static summarization graphs. Each one of these applications goes through the same development phases. Figure 1 shows the most common phases that an application would go through. First, the text data needed by the system must be collected. All the explored applications in this work use the CORD19 [7] dataset (either a version of it or a subset of a version of CORD12). Second, the raw data collected may need to be transformed in some cases to meet certain specifications. This can be achieved by enriching the data in order to make it more representative, or it can be achieved by structuring the available data differently. Third, given the available data and the basic application specifications, a set of learning problems (i.e., question answering, document retrieval, passage retrieval) need to be defined. Forth, given the defined learning problem, machine learning models are developed and trained to achieve the learning tasks. Fifth, the models are evaluated, either by a human or an automated evaluation process. Sixth, after evaluating the models, they need to be deployed to ensure their accessibility by a larger number of users, and that is by providing an easy-to-use user interface with a reliable model execution backend architecture.

Although a previous survey [9] has explored the COVID-19 literature search engines, their work has certain limitations that we try to remedy in this work. First, rather than focusing primarily on textual search engines, we explore visual search engines. Second, ref. [9] included a plethora of applications that are not associated with any research papers or technical reports. Consequently, we discarded these applications and focused on applications with research papers in order to gain and express a deeper understanding of the methods that they employed. Third, we try to infer some design principles that the authors of the works used to create their system.

This work is organized as follows: In Section 2, we describe some datasets that were used in the explored works for various purposes. In Section 3, we explore the characteristics and design principles of the COVID-19 exploratory literature search applications. In Section sec:eval, we explore certain methods that were used to evaluate the systems. In Section 5 we show certain limitations of the examined works. Section 6 concludes our work, and the following section (Section 7) gives certain limitations that this work has.

## 2. Datasets

In this section, we list some of the datasets that were used in the works that we explored. We categorized the datasets relative to their structure into three categories: (a) unstructured, (b) structured and (c) hybrid.

### 2.1. Unstructured Datasets

Unstructured data are information that does not have a defined data model. This type of data is mainly textual in nature. The following structured and hybrid datasets have been built using unstructured data. In fact, all the previously mentioned categories were either automatically or manually curated and annotated from different literature databases (e.g., Arxiv, DBLP, Pubmed, bioRxiv, medRxiv), which contain unstructured documents, often in a hard-to-read format, such as PDFs.

### 2.2. Structured Datasets

We can recognize two kinds of structured data: (a) data with tabular structure, where every example shares the same set of variables and examples are independent of each other, and (b) data with relational structure, where examples do not necessarily share the same set of variables, examples are inherently typed; that is, each example belong to a predefined group of examples, and examples have a dependency between them, which is implemented practically in the form of links.

The first category contains mainly annotated datasets that are oriented for machine learning purposes, such as training, fine-tuning, or evaluating the created models on specific tasks. The works that we explored use multiple datasets. A later section defines some of the main tasks that the works try to solve. All of these tasks are text-oriented and can fall under the umbrella of information retrieval in general. Annotated datasets such as TREC-COVID [8] and BioASQ [10] were used for document retrieval. These datasets are generally constructed by a set of human curators who were provided with a list of queries (or questions) and a set of supposedly relevant documents, and the goal was to select the most pertinent documents for each query. In addition, multiple datasets have been used to train question answering models such as COVIDQA [11], COVID-19 Questions [12], COVID-QA [13], InfoBot Dataset [14], MS-MARCO [15], Med-MARCO [16], Natural Questions [17], SQuAD [18], BioASQ [10], M-CID [19] and QuAC [20]. Other datasets were used to train document summarization models. For example, DUC 2005 [21], 2006 [22] and Debatepedia [23] were used by [24] to train document summarization models. Other datasets, such as GENIA [25], JNLPBA [26], CHEMDNER [27], NCBI Disease Corpus [28], CHEMPROT [29], BC5CDR [30] and COV19_729 [31], were used for the named entity recognition (NER) of multiple types of entities, namely, chemicals, genes, proteins, diseases and other biomedical entities. Relation extraction (RE) was also a task of interest in [31], which was achieved using the CHEMPROT [29] and BC5CDR [30] datasets. NER and RE tasks are generally used in knowledge graph construction, where the entities extracted represent nodes, and the relations represent edges between nodes. Some of these datasets were curated using data from COVID-19 related source documents, e.g., COVIDQA [11], COVID-19 Questions [12], COVID-QA [13], InfoBot Dataset [14] and TREC-COVID [8]. Table 1 summarizes the the previously mentioned datasets.

Concerning data with relational structure, some works used knowledge graphs constructed from the COVID-19-related literature. In general, the graphs contain four types of entities with multiple properties: (1) a paper entity, which represents a research paper and can be described by a Digital Object Identifier (DOI), title, publication date and other properties; (2) an author entity, which represents a publication’s author, and can be described by an identifier, a first, middle and last name and other properties of interest; (3) an affiliation entity, which represents the research structure (lab, university, company, etc.) to which the author is affiliated, which can be described by an identifier, a name and other properties of interest; (4) a concept entity, which represents a domain knowledge-related notion that exists in a paper. A concept can be represented by one word or a series of words. Concepts can have multiple types of relationships between them, depending on the type of concepts. For example, concepts of biomedical types, such as genes, diseases, chemicals, organisms and proteins, can be linked by semantic biomedical relationships [31,32,33,34] or by syntactic relationships based on their co-occurrence in the same sentence [35]. Table 2 and Table 3 offer a more detailed description and these entities and how they are related. Table 4 represents multiple KGs and their description. It is worth pointing out that not all knowledge graphs respect this schema. Some implement it totally (e.g., CKG [36]), and some implement it partially (e.g., CovEx KG [37]), as shown in Table 2 and Table 3.

Furthermore, it has been observed that the design of certain knowledge graphs is dependent on the tasks they are used for. For instance, for the task of document retrieval, a knowledge graph is generally designed with documents as the central nodes to which other nodes may be linked [36,37]. On the other hand, for the task of question answering, even though the same base data is used, no node holds the document data; instead, documents are ignored, and only concept nodes are presented and interlinked [34]. In addition, the granularity of the relationships and the entities are also important, as it was demonstrated in [33,34], where two types of relationships and entities were extracted: (a) coarse-grained and (b) fine-grained. The latter was needed in a question-answering task to accommodate the specificity of the entities expressed in user queries, which is not required in other tasks, as shown in [31] for the task of link prediction, where the authors discarded fine-grained relationships in favor of more general ones to reduce noise that can hinder the performance of certain models. In the case of network visualization, ref. [35] adopted a more flexible approach to KG construction by extracting a set of entities and saving them so that they could be later aggregated to create domain-specific networks, which can be visualized. Some tasks, such as information extension, which aims at enriching certain information constructs such as queries or KGs, do not need directed edges, which is the case, for example, in Vapur KG [32] and Citation KG [44]. In fact, having undirected edges help explore more complex and unexpected relationships among entities, which was illustrated in a fact-checking application in [45].

### 2.3. Hybrid

Hybrid datasets have some structure, which can be in the form of tags, but most if not all of the tagged elements have no structure, which generally means that these elements are in a textual format. An example of such datasets is CORD19. The CORD-19 dataset is the centerpiece of the COVID-19 literature exploration applications. The CORD-19 dataset [7] is a curated set of articles from multiple resources that were collected to help efforts against the COVID-19 pandemic. This dataset was used in a common document retrieval task TREC-COVID, where a set of CORD-19 articles were curated and annotated for their relevance relative to certain user queries. The dataset is ever-expanding, with new articles being added to it intermittently. The dataset is available online at (as of in 4 April 2022) https://www.kaggle.com/allen-institute-for-ai/CORD-19-research-challenge.

## 3. Exploratory Search Applications

### 3.1. Textual Exploratory Search

**Figure 2:**
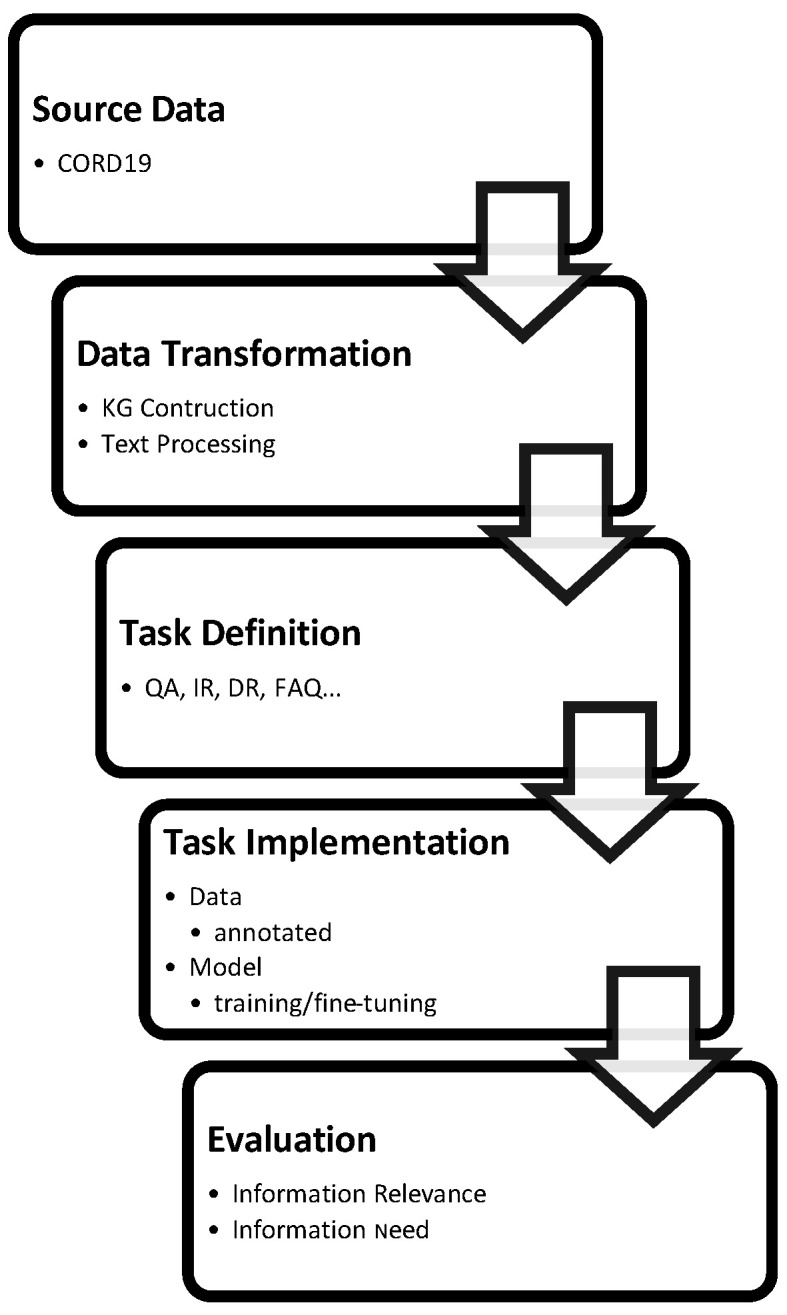
Summary of The Development Process of Literature Search Engines.

Research related to COVID-19 knowledge management and information retrieval (KM&IR) has gained tremendous attention over the past year. Here, we try to present a concise summary of the research in this area. The development of search engines goes through certain common steps that are illustrated in Figure 2. A search engine’s development process begins with the base data or the data that are relevant to the search query. Second, the raw textual data are processed to extract certain elements that are of interest and transform them. That same raw data can be reorganized in the form of a knowledge graph to satisfy certain specifications such as fast question answering. Afterward, the tasks that are intended for the search engine should be defined and implemented, followed by an assessment of the efficiency of the system in performing those tasks. Finally, the implemented system needs to be deployed for public access.

COVID-19 literature knowledge management and information retrieval systems have multiple axes along which we can study, survey and compare them. We list some of these characteristics in what follows:

- **Tasks**: The tasks are related to textual data, and hence we suppose that we have a text database (or collection or corpus) T as a string of *N* symbols drawn from an alphabet (i.e., all possible combinations of letters) Σ. A vocabulary *V* is the set of unique words used in T. T is partitioned into *n* documents $\left\{d_{1},d_{2},\ldots ,d_{n}\right\}$. A document *d* can be presented as $\left(w_{d,1},w_{d,2},\ldots ,w_{d,n_{d}}\right)$ in T, including $n_{d}$ words from *V*. Queries are also strings (or sets of strings) composed of symbols drawn from Σ. Symbols in Σ may be letters, bytes or even words, and the documents may be articles, chromosomes or any other texts in which we need to search. In general, these tokens are extracted using tokenizers and further processed using lemmatization, stemming and other techniques that help normalize tokens. In the explored systems, we can identify the following tasks.
  − *Document Retrieval (Indexing, Ranking): for this task, two sub-tasks can be* identified [3].
    1. Document Listing: given query $Q=\{q_{1},\ldots ,q_{m}|q_{i}\in \Sigma^{*},\forall i\}$ and a text $T\in \Sigma^{*}$ that is partitioned into *n* documents, $\left\{d_{1},d_{2},\ldots ,d_{n}\right\}$, the aim of this task is to return a list of the documents in which one or multiple tokens of *Q* appear at least once.
    2. Document Ranking: given a query $Q=\{q_{1},\ldots ,q_{m}|q_{i}\in \Sigma^{*},\forall i\}$, an integer 0<k≤N, and a text $T\in \Sigma^{*}$ that is partitioned into *n* documents $\left\{d_{1},d_{2},\ldots ,d_{n}\right\}$, and returns the top-*k* documents ordered by a similarity measure $S(Q,d_{i})$.
  − *Passage Retrieval (Indexing, Ranking)*: Given a query *Q*, and a set of documents *D* where each document is partitioned into passages, the aim of this task is to find relevant passages for the query [5]. Passage retrieval can also be used for sentence highlighting.
  − *Question Answering*: Given a Query $Q=\{q_{1},q_{2},\ldots ,q_{m}\}$ made of *m* tokens and a passage $P=\{p_{1},p_{2},\ldots ,p_{k}\}$ made of *k* tokens, the aim of this task is to find an answer span $A=\{a_{\mathrm{start}},a_{\mathrm{end}}\}$ in *P* [4].
  − *Summarization*: We will opt for the definition presented in [6]. Given a set of documents $D=d_{i}$ that we will call source documents, summarization aims to generate a text *s* (called summary) that is coherent and contains a significant amount of relevant information from the source text. Ref. [6] considered a good summary to have a compression rate $\tau =\frac{c(s)}{c(D)}$ (where c(x) is the word count in *x*, *x* can be a sentence or document or any grouping of words) of less than a third of the length of the original document.
  − *Topic Modeling*: The aim of topic modeling is to infer a set of *K* topics capturing a lower-dimensional representation suitable for summarization and prediction tasks [46]. According to [47], Given a text corpus T with a vocabulary of size *V* and the predefined number of topics *K*, the major tasks of topic modeling can be defined as:
    1. Learning the word representation of topics α: a topic α in a given collection T is defined as a multinomial distribution over the vocabulary *V*, i.e., ${p(w|\alpha )}_{w\in V}$.
    2. Learning the sparse topic representation of documents θ: the topic representation of a document *d*, $\theta_{d}$, is defined as a multinomial distribution over *K* topics, i.e., ${p(\alpha_{k}|\theta_{d})}_{k=1,\ldots ,K}$.
    In general, the task of topic modeling aims to find *K* salient topics $\alpha_{k=1,\ldots ,K}$ from T and to find the topic representation of each document $\theta_{d=1,\ldots ,n}$.
  *FAQ Matching*: let *F* denote the set of question–answer pairs; given *F* and a user query *Q*, this task aims to rank the question–answer pairs in *F*. The top *k* QA pairs with high scores are returned to the user [48].
  *Recommendation*: Given the set of all users C and the set of all possible items that can be recommended S. Let *u* be a utility function that measures the usefulness of item *s* to user *c*, i.e., u:C×S→R, where R is a totally ordered set (e.g., non-negative integers or real numbers within a certain range). The goal of this task is to choose the item(s) s∈S that maximize(s) the utility for each user c∈C [49].
- **Feedback Loop**: this characteristic is related to the use of user feedback data in any of the mentioned tasks.
- **Representation Level for Text**: In general, text can be represented in two distinct spaces: (a) bag-of-words space, (b) vector space. These representations can be shown on one or multiple levels of granularity of textual documents; that is, *Document Level*, *Paragraph Level*, *Sentence Level* and *Word Level*.
- **Representation Levels for Graphs**: Graphs can also be represented in a frequentist space or low-dimensional vectorial space. These representations can be shown on one or multiple levels of granularity of graphs; that is, *Full Graph Level*, *Sub-graph Level*, *Node Level* and *Edge Level*. Examples of graph representation in COVID-19 literature search engines are as follow:
  − Document Sub-graph Embedding: in order to make document-level embeddings, refs. [36,38] combined document-level textual embeddings with embeddings of documents’ related sub-graphs from the bigger KG to recommend similar documents.
- **Novelty**: a research paper is said to have novelty if the authors explored uncharted territories to solve old or new problems. Specifically, we characterize papers to have novelty if they contain new contributions to the design of models, learning objectives or data processing. We ignored the data aspect of this characterization because all the papers can be considered to be novel considering only data.
- **Data Enrichment**: Data enrichment refers, in general, to the process of adding more data to the already existing training data. Data enrichment methods can take two main forms, (a) data augmentation and (b) data supplementation. The former is characteristic of the set of methods that use the already existing data to generate more data, while the latter encapsulates methods that use external resources in order to supplement the available data. The latter is easy to accomplish as long as there are external resources. There are various data augmentations methods. For example, in CO-Search [39], in order to train a Siamese network, the authors generated negative (*paragraph*, *reference*) pairs based on positive pairs extracted from documents.
- **Search Type**:
  − *Keyword*: Keyword search refers to searching using queries composed of one specific word.
  − *Regular Expression*: In this type of search, the query takes the form of regular expressions that annotates textual patterns that we would like to retrieve. For example, ref. [50] used this search strategy to look for drugs with certain properties in a drug re-purposing database.
  − *Open Questions*: This type of search refers to using natural language queries with simple or complex structures.
  − *Keyphrase Search*: This type of search refers to using queries composed of one or multiple keywords, and the order is taken into consideration.
- **KG Traversal**: This refers to the use of knowledge graphs to search for entities or relationships that are relevant to achieving one or multiple tasks.
- **Representation Combination (Rep.Comb.)**: This characteristic exists in one of two cases: (a) the combination of multiple levels of representation to achieve a task, or (b) the combination of KG and textual representation to achieve a task.

Table 5 offers an exhaustive list of search engines and their design specifications. While exploring search engines for the COVID-19 literature, we noticed multiple characteristics that are elaborated on in what follows:

- **Fast Prototyping and Deployment**: Given the urgent nature of most of the applications, the researcher opted mainly for off-the-shelf technologies that are easy to work with. In addition, except for one application, all the other applications used existing models and algorithms, which can also be attributed to the urgency of the task.
- **Textual Representation Methods**: There are two categories of methods: (a) Bag-of-Words (BOW) models and (b) Vector Space Models (VSMs). The major difference is that VSMs capture more of the contextual elements of text than the BOW methods, but on the other hand, the VSMs are computationally more expensive during training and inference. Some works struck a balance by applying both categories of methods, e.g., [37,44,51,52], which is performed generally by using a multi-stage ranking scheme that applies the first ranking using BOW models, which is then followed by a re-ranking using a VSM of the output of the previous ranking. Some works compensate for the latency of neural language models [12] by pre-indexing documents offline.
- **Granularity/Levels of Representations**: We also noticed that the works used different levels of granularity, which depends on the intended tasks and the available computational resources. For example, to achieve the task of document retrieval, some works opted for simple document level representations [53], while other works either used more granular representations [12,32,37,40,50,54,55,56] or a mix of more granular representations with document level representations [16,24,38,39,44,51,52,57].
- **Using KGs**: Knowledge graphs were used in multiple works for different purposes. For example, ref. [38] used a KG (**CKG** [36]) embedding in tandem with textual representations for document recommendation, while [37] (**CovEx KG** [37]), [32] (**Vapur KG** [32]) and [44] (**Citation KG** [44]) traversed their respective KGs looking for similar entities to retrieve relevant papers. The authors of [56] (**Blender-KG** [34]) used a KG to extend queries and make the search more efficient.
- **Recommendation Modules**: Many search engines [32,37,38] use recommendation modules to offer more user-oriented results.
- **Query Transformation/Extension**: Query transformation is also used in many applications to make the queries more expressive, which can help get more relevant results. For example, ref. [53] used an extensive database of medical terms to augment the queries made by novices to search an academic biomedical corpus.
- **Multimedia (e.g., image, video, etc.) Grounding**: Multimedia grounding is also used to couple textual data with relevant multimedia content. For example, ref. [54] used a self-supervised method to couple biomedical text with corresponding coordinates in a human atlas. This mapping was used to conduct two kinds of queries: (a) atlas-based document retrieval using textual queries (which contain mentions of body parts) and (b) atlas-based document retrieval using 3D atlas coordinates. In addition, ref. [34] associated figures that depict molecular structures in research papers with their chemical entities that exist in a KG by using the captions of the figures. This was done to augment the KG.

### 3.2. Visual Exploratory Search

While exploring the COVID-19 literature, researchers can face two kinds of challenges: (a) quantity of the research papers and (b) the quality of the research papers. Even though the textual exploratory search is a useful literature exploration tool, it is targeted and requires the researcher to know what she/he is looking for in advance, which is not always evident. Consequently, many visual exploratory search tools have been developed to explore the COVID-19 literature in a visual, interactive and general manner, rather than having to go through the tedious process of manually curating the literature. In the context of scientific literature, this can also be used to explore latent structures within the data which may be related to co-authorship networks, citation networks and other important bibliometric dimensions.

**Figure 3:**
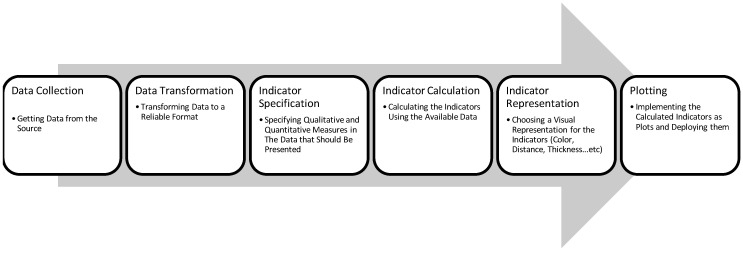
Summary of Exploratory Search Application Creation Process.

In light of the reviewed literature, we can infer a general process that exploratory visual search applications follow. This process is presented in Figure 3. The most important two phases of this process are (a) indicator specification and (b) indicator representation. The former is where one or multiple quantitative (e.g., entity types, topics, affiliation, etc.) or qualitative characteristics (e.g., occurrence/co-occurrence frequency/count) of the data are chosen to be presented, and their method of presentation is also specified. The latter phase is where a significant visual representation is chosen for those indicators; for example, qualitative indicators can be presented using colors, and quantitative indicators can be presented using distance, surface or volume variations.

**Figure 4:**
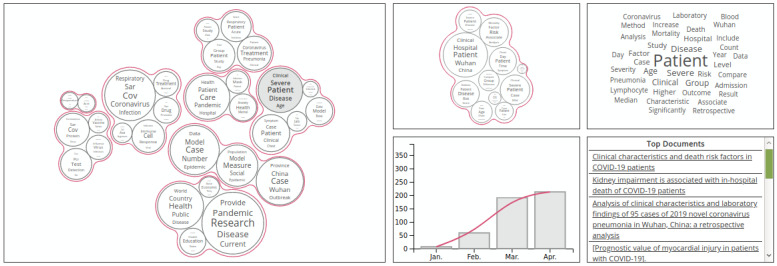
TopicMaps Interface.

**Figure 5:**
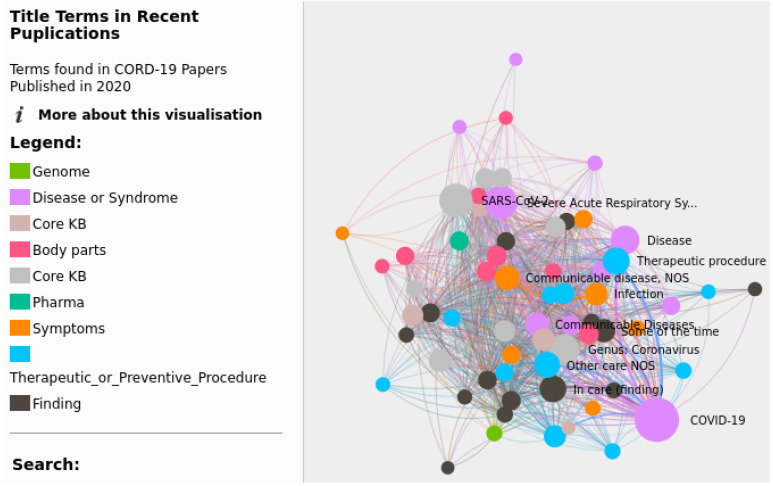
Network Visualization Interface.

The data used for the exploratory search applications are either CORD-19 [7] or one of the knowledge graphs presented previously. The frequency and count indicators are the most predominantly used, although other indicators are also used. For example, ref. [58] uses topic similarity vectors to cluster similar topics. Multiple plots and visualization tools were used to visualize the indicators (see Figure 4); these are summarized in Table 6. In addition, some works use certain tasks in the data transformation phase in order to get more relevant data from the raw text. The tasks mentioned in the works are information extraction (IE), which is generally attributed to basic textual information extraction, topic modeling, which was used in [58], and NER, which was used in [35,43,59] to extract named entities and use their count as an indicator, and network analysis [35,59]. In [35], network analysis was used to solve two problems faced during network traversal, namely the problem of network size and the search for deep connections, using a breadth-first-search technique on the network structure. In [35], network analysis was used to detect communities within a co-authorship network, motivated by the need to keep track of what other groups were doing in order to explore new fields and potential collaborations. Figure 5 shows the interface proposed by [35]. Reactivity is also an important feature in these tools since it simplifies interactive visual manipulation, which makes the exploration more flexible. Public availability is also looked into, and links to the tools are provided if they exist.

## 4. Evaluation Methods

In general, machine learning models are composed of two main modules, (a) a representation module and (b) a decision module. The former is responsible for transforming the data from a complex multidimensional space with latent spatial and temporal dependencies to a lower-dimensional and more abstract space. The second module is used to process the representational modules’ output to achieve a task. The training of these modules can be performed independently; that is, the representational module can be trained separately in an unsupervised or self-supervised manner, while the combination of the two modules can be trained in a self-supervised, semi-supervised or fully supervised manner.

The machine learning (ML) models used in the previously explored works, be it search engine-related ML models or knowledge graph creation ML models (e.g., named entity recognition models), have to be evaluated to get empirical evidence on their viability. While exploring the literature, we noticed that there are two main evaluation techniques: human evaluation and automatic evaluation. The former bases its evaluation on the relevance judgment of the users, and the latter focuses on information needs in order to evaluate the results. The latter also has two sub-categories of evaluation measures: intrinsic evaluation measures and extrinsic evaluation measures.

### 4.1. Human Evaluation

Human evaluation is based on quantifying human feedback towards the evaluated application. This type of evaluation is advantageous because of its integral character. Indeed, humans can evaluate more complex applications with multiple interacting modules. For example, in the case of a search engine, a human evaluator can assess the information relevance of the search results in addition to some representational aspects, such as highlighting, which are not easy to evaluate automatically [51,57]. However, the downside of the human evaluation method is its irreplicability due to the fact that human evaluation is inherently biased and depends on the needs that the evaluators have, their field of expertise and what they expect from the application. For example, an experienced researcher may find longer spans of text more reliable as answers to a query, while a novice would generally prefer direct short answers [40]. This makes performance comparison of multiple applications based on human evaluation generally unreliable.

### 4.2. Automatic Evaluation

Automatic evaluation is the de facto evaluation method in the machine learning literature. It is based on using evaluation metrics that quantify the discrepancy that exists between the model output and the wanted output. This is advantageous since it puts multiple applications on an equal footing during evaluation, which is advantageous. On the other hand, automatic evaluation is monolithic, meaning that it only evaluates one aspect of an application at a time (e.g., QA, DR, IR, etc.) and not the integrality of the application as is the case in human evaluation [51,57]. Furthermore, some aspects, such as ease of use and interface interactivity, cannot be evaluated automatically. In addition, the evaluation metrics used can suffer from certain biases that can lessen the validity of the evaluation. For example, ref. [40] has found that automatic metrics such as F1 heavily penalize long answers, as they overlap poorly with the gold annotations, which are mostly short, factual answers.

As was mentioned before, automatic evaluation measures can be categorized into (a) intrinsic evaluation measures (IEMs) and (b) extrinsic evaluation measures (EEMs). The former measures are generally used to evaluate representation modules separately, and the latter measures are used to evaluate the combined representation and decision downstream model.

#### 4.2.1. Intrinsic Evaluation

In the explored works, we only found one example of intrinsic evaluation [31], where KG node embeddings are evaluated by comparing the Pearson and Spearman correlation scores between the ratings and the cosine similarity scores of entities.

#### 4.2.2. Extrinsic Evaluation

In contrast to IEM, EEMs are more frequently used. The works that we explored use a plethora of EEMs that depend on the kind of tasks to be evaluated. This type of evaluation is performed through multiple evaluation metrics that are task-specific. Multiple evaluation measures and their variants were used. For example, the ROUGE evaluation metric [61] and its variants were used in [24] to evaluate the summarization models. The Match method [62,63] was used in [41] to evaluate QA and IR. Other more-standard evaluation metrics such as recall and precision were used for IR tasks [64].

## 5. Discussion and Future Research Directions

In general, the explored works have certain common limitations. In what follows, we summarize a few of them:

- **Evaluation**: Most of the applications (e.g., [40,51,57]) suffer from a monolithic evaluation scheme that focuses on one task in particular and ignores other aspects of the application, especially those related to visual aspects.
- **Feedback Loop**: Some applications (e.g., [51,57]) expressed the importance of including human input in the process of information retrieval, as it tends to balance information need and information relevance.
- **Fact Checking**: Due to the rapid expansion of the COVID-19 literature and the existence of many contradictory claims concerning, for example, the incubation period of the virus and the optimal social distancing protocol stresses the importance of fact checking applications for COVID-19 claims. The authors of [65] created a claim verification application for the COVID-19 literature, which uses a passage and a claim as input and outputs if the claim is true or not given the passage. This type of application needs huge amounts of annotated data, which is particularly cumbersome in the case of COVID-19 since it needs skilled specialists to annotate it. Developing semi-supervised or unsupervised techniques would be useful.
- **Extending Data**: Most of the applications (e.g., [53,54]) used limited amounts of data (labeled or not) to perform tasks, either because of the lack of labeled data or because of the lack of computational resources. More data would certainly improve performance.
- **Data Bias**: Some applications (e.g., [54]) can also benefit from reducing data bias, especially gender bias.
- **Smart Querying**: Some applications [56] use query functionalities that tend to be limited to simple word matching. This can be problematic in cases where the intent of the user is not evident in the query. This can be remedied by using embedding-based query matching, which uses contextual information for matching the queries to the results.

## 6. Conclusions

This work represents an exploration of COVID-19 literature exploration applications, with emphasis on their design principles and concepts. There are two main types of literature exploration applications, (a) exploratory textual search and (b) exploratory visual search. The former uses textual queries made by end-users in order to explore the knowledge base and send the most relevant documents back to the users, while the latter type of application uses visual summaries to offer a structured view of the existing literature.

## 7. Limitations of This Work

Empirical quantitative evaluation of the systems explored in this work was of interest, but discrepancies were found in the evaluation results of the same systems in multiple sources (e.g., the results given in [38] are different from those given in [51] for the same system: COVIDex), in addition to the unavailable implementation details of some systems, discouraged us from pursuing the objective in this work.

## Figures and Tables

**Table 1:** Summary of the Datasets. NER refers to Named Entity Recognition, RE refers to Relationship Extraction, SMZ refers to summarization, QA refers to Question Answering, DR refers to Document Retrieval.

Dataset	Application Refs	Tasks	Statistics	URL
TREC-COVID [8]	[16,38,39,40]	DR	The TREC-COVID dataset has many versions which correspond to TREC-COVID challenges. For example, round three contains a total of 16,677 unique journal articles in CORD-19 with a relevance annotation.	https://www.kaggle.com/c/trec-COVID-information-retrieval/data (accessed on 4 April 2022)
COVIDQA * [11]	[24,38,41]	QA	The dataset contains 147 question–article–answer triples with 27 unique questions and 104 unique articles.	https://github.com/castorini/pygaggle/tree/master/data (accessed on 4 April 2022)
COVID-19 Questions * [12]	[12,41]	QA	The dataset contains 111 question–answer pairs with 53 interrogative and 58 keyword-style queries.	https://drive.google.com/file/d/1z7jW0fovgTfTScCanZvrvrUax1HAMEFV/view?usp=sharing (accessed on 4 April 2022)
COVID-QA * [13]	[40,41]	QA	The dataset consists of 2019 question–article–answer triples.	https://github.com/deepset-ai/COVID-QA (accessed on 4 April 2022)
InfoBot Dataset * [14]	[42]	QA, FAQ	2200 COVID-19-related Frequently asked Question–Answer pairs.	https://covid-19-infobot.org/data/ (accessed on 4 April 2022)
MS-MARCO [15]	[16]	QA	1,000,000 training instances.	https://microsoft.github.io/msmarco/ (accessed on 4 April 2022)
Med-MARCO [16]	[16]	QA	79K of the original MS-MARCO questions (9.7%).	https://github.com/Georgetown-IR-Lab/covid-neural-ir/blob/master/med-msmarco-train.txt (accessed on 4 April 2022)
Natural Questions [17]	[12]	QA	The public release consists of 307,373 training examples with single annotations; 7830 examples with 5-way annotations for development data; and a further 7842 examples with 5-way annotated sequestered as test data.	https://ai.google.com/research/NaturalQuestions/ (accessed on 4 April 2022)
SQuAD [18]	[12]	QA	The dataset contains 107,785 question–answer pairs on 536 articles.	https://rajpurkar.github.io/SQuAD-explorer/ (accessed on 4 April 2022)
BioASQ [10]	[12]	QA, DR	500 questions with their relevant documents, text span answers and perfect answers.	http://www.bioasq.org/news/golden-datasets-2nd-edition-bioasq-challenge-are-now-available (accessed on 4 April 2022)
M-CID [19]	[19]	QA	The dataset is composed of 6871 natural language utterances across 16 COVID-19-specific intents and 4 languages: English, Spanish, French and German.	https://fb.me/covid_mcid_dataset (accessed on 4 April 2022)
QuAC [20]	[40]	QA	14K information-seeking QA dialogs, and 100K questions in total.	http://quac.ai/ (accessed on 4 April 2022)
GENIA [25]	[37]	NER	2000 abstracts taken from the MEDLINE database; contains more than 400,000 words and almost 100,000 annotations.	http://www.geniaproject.org/genia-corpus/term-corpus (accessed on 4 April 2022)
DUC 2005, 2006 [21,22]	[24]	SMZ	The dataset is composed of 50 topics.	https://www-nlpir.nist.gov/projects/duc/data.html (accessed on 4 April 2022)
Debatepedia [23]	[24]	SMZ	It consists of 10,859 training examples, 1357 testing and 1357 validation samples. The average number of words in summary, documents and query is 11.16, 66.4 and 10, respectively.	https://github.com/PrekshaNema25/DiverstiyBasedAttentionMechanism (accessed on 4 April 2022)
JNLPBA [26]	[31]	NER	This dataset contains a subset of the GENIA dataset V3.02. This subset is composed of 2404 abstracts. The articles were chosen to contain the MeSH terms “human”, “blood cells” and “transcription factors”, and their publication year ranges from 1990 to 1999.	http://www.geniaproject.org/shared-tasks/bionlp-jnlpba-shared-task-2004 (accessed on 4 April 2022)
CHEMDNER [27]	[31]	NER	10,000 PubMed abstracts that contain a total of 84,355 chemical entities.	https://biocreative.bioinformatics.udel.edu/resources/biocreative-iv/chemdner-corpus/ (accessed on 4 April 2022)
NCBI Disease Corpus [28]	[31]	NER	793 PubMed abstracts that were annotated. A total of 6892 disease mentions, which are mapped to 790 unique disease concepts that were extracted.	https://github.com/spyysalo/ncbi-disease (accessed on 4 April 2022)
CHEMPROT [29]	[31]	NER, RE	2500 PubMed abstracts, from which 32,000 chemical entities and 31,000 protein entities were extracted. In addition, 10,000 chemical-protein relationships were extracted.	http://www.biocreative.org/accounts/login/?next=/resources/corpora/chemprot-corpus-biocreative-vi/ (accessed on 4 April 2022)
BC5CDR [30]	[31]	NER, RE	1500 PubMed articles with 4409 annotated chemicals, 5818 diseases and 3116 chemical-disease interactions.	https://github.com/shreyashub/BioFLAIR/tree/master/data/ner (accessed on 4 April 2022)
COV19_729 * [31]	[31]	NER	The dataset is composed of 729 examples. Each example is a triple comprising an entity, the class that that entity belongs to (i.e., disease, protein, chemical), and a physician’s rating of how related those entities are to COVID-19.	https://github.com/sayantanbasu05/ERKLG (accessed on 4 April 2022)

**Table 2:** Examples of Entities Specifications.

Entities	Properties	Description	ID
Paper	title, publication date, journal, Digital Object Identifier (DOI), link	Representation of research paper entities.	E1
Author	identifier, first names, middle names, last names	Representation of the paper authors.	E2
Affiliation	identifier, name, country, city	Representation of a research structure where an author belongs.	E3
Concept	concept identifier, textual value, concept type (gene, disease, topic, chemical, etc.)	Representation of a domain specific concept.	E4

**Table 3:** Examples of Relations.

Source Entity	Dest. Entity	Relation	Description	ID
Paper	Paper	cites	This relation connects paper entities with paper references indicating a citation relation.	R1
Author	Author	co-author	This relation connects an author entity with another author entity indicating a co-authorship relation.	R2
Concept	Concept	relate concepts	This relationship links two concepts with any general relationship that might link them.	R3
Paper	Author	authored by	This relation connects paper entities with author entities and indicates an authorship relation.	R4
Paper	Concept	associated concept	This relation connects paper entities with concept entities.	R5
Author	Affiliation	affiliated with	This relation connects author entities with institution entities.	R6
Author	Concept	research area	This relation connects author entities with concept entities indicating a research area of the author.	R7

**Table 4:** Summary of Knowledge Graphs Related to COVID-19.

KG	Usage	Ent.	Rel.
CKG [36]	Article recommendations, citation-based navigation, and search result ranking.	E1, E2, E3, E4	R1, R4, R6, R5
CovEx KG [37]	Document Retrieval.	E1, E2, E4	R1, R4, R5, R7
ERLKG [31]	Link prediction.	E4	R3
COVID-KG [34] (Blender-KG [43])	QA, Semantic Visualization, Drug Re-purposing.	E4	R3
COFIE KG [33]	KG search over relations and entities using a query.	E4	R3
Network Visualization KG [35]	Data Visualization.	E4	R3
Vapur KG [32]	Query extension.	E4	R3
Citation KG [44]	Document Ranking.	E1	R1

**Table 5:** Search Engine Comparison. “❙” signifies the existence of the corresponding characteristic, and “✓” signifies the lack of it. Marks between parentheses correspond to characteristics between parentheses.

System		CO-Search [39]	AWS CORD-19 Search (ACS) [38]	COVID-19 Drug Repository [50]	CovEx [37]	COVIDex [51,57]	Vapur [32]	COVIDASK [12]	[40]	CAiRE-COVID [24]	[41]	CORD19-Explorer [54]	SLEDGE-Z [16]	S_COVID [52]	[44]	SLIC [53]	SPIKE [55]	EVIDENCEMINER [56]
Uses Raw Text (Uses KG)		✓(❙)	✓(✓)	✓(❙)	✓(✓)	✓(❙)	✓(✓)	✓(❙)	✓(❙)	✓(❙)	✓(❙)	✓(❙)	✓(❙)	✓(❙)	✓(✓)	✓(❙)	✓(❙)	✓(✓)
Publicly Available		✓	✓	✓	✓	✓	✓	✓	❙	✓	❙	✓	❙	✓	❙	❙	✓	✓
Feedback Loop		❙	❙	❙	✓	✓	❙	❙	❙	❙	❙	❙	❙	❙	❙	❙	❙	❙
Multistage Ranking		❙	❙	❙	✓	✓	❙	❙	❙	❙	❙	❙	❙	✓	✓	❙	❙	❙
KG Traversal		❙	✓	❙	❙	❙	✓	❙	❙	❙	❙	❙	❙	❙	✓	❙	❙	✓
Text Representations Levels (KG Representation Level)	Document (KG)	✓(❙)	✓(❙)	❙(❙)	❙(❙)	✓(❙)	❙(❙)	❙(❙)	❙(❙)	✓(❙)	❙(❙)	❙(❙)	✓(❙)	✓(❙)	✓(❙)	✓(❙)	❙(❙)	❙(❙)
	Paragraph (Sub-graph)	✓(❙)	✓(✓)	❙(❙)	❙(❙)	✓(❙)	❙(❙)	❙(❙)	✓(❙)	✓(❙)	✓(❙)	❙(❙)	✓(❙)	❙(❙)	❙(✓)	❙(❙)	✓(❙)	❙(❙)
	Sentence (Edge)	✓(❙)	❙(❙)	❙(❙)	❙(❙)	❙(❙)	❙(❙)	✓(❙)	❙(❙)	✓(❙)	✓(❙)	✓(❙)	❙(❙)	✓(✓)	✓(❙)	❙(❙)	✓(❙)	✓(❙)
	Word (Node)	❙(❙)	❙(❙)	❙(❙)	✓(❙)	❙(❙)	❙(❙)	❙(❙)	❙(❙)	❙(❙)	❙(❙)	❙(❙)	❙(❙)	✓(❙)	❙(❙)	❙(❙)	✓(❙)	✓(❙)
	n-gram (Node Property)	❙(❙)	❙(❙)	❙(❙)	❙(❙)	❙(❙)	✓(❙)	❙(❙)	❙(❙)	❙(❙)	❙(❙))	❙(❙)	❙(❙)	❙(❙)	❙(❙)	❙(❙)	❙(❙)	❙(❙)
	Keyphrase (Edge Property)	❙(❙)	❙(❙)	✓(❙)	❙(❙)	❙(❙)	❙(❙)	❙(❙)	❙(❙)	❙(❙)	❙(❙)	❙(❙)	❙(❙)	❙(❙)	❙(❙)	❙(❙)	✓(❙)	✓(❙)
Rep.Comb.	Inter-Level	✓	❙	❙	❙	✓	❙	❙	❙	✓	❙	❙	✓	✓	✓	N❙	✓	❙
	Text & KG	❙	✓	❙	❙	❙	❙	❙	❙	❙	❙	❙	❙	❙	✓	❙	❙	❙
Tasks	Document Retrieval (Indexing, Ranking)	✓	✓	✓	✓	✓	✓	✓	✓	✓	❙	✓	✓	✓	✓	✓	✓	✓
	Passage Retrieval (Indexing, Ranking)	✓	✓	❙	❙	✓	❙	✓	✓	✓	✓	❙	❙	✓	✓	❙	✓	✓
	Question Answering	✓	✓	❙	❙	✓	❙	✓	✓	✓	✓	❙	❙	❙	✓	❙	❙	❙
	Summarization	✓	❙	❙	❙	❙	❙	❙	❙	✓	❙	❙	❙	❙	✓	❙	❙	❙
	Topic Modeling	❙	✓	❙	✓	❙	❙	❙	❙	❙	❙	❙	❙	✓	✓	❙	❙	❙
	Recommendation	❙	✓	❙	✓	❙	✓	❙	❙	❙	❙	❙	❙	❙	❙	❙	❙	❙
	FAQ Matching	❙	✓	❙	❙	❙	❙	❙	❙	❙	❙	❙	❙	❙	❙	❙	❙	❙
Search Type	Keyword	❙	✓	✓	✓	✓	✓	✓	❙	✓	❙	✓	❙	✓	✓	✓	✓	✓
	Open Questions	✓	✓	❙	❙	❙	❙	✓	✓	✓	✓	❙	✓	✓	✓	✓	❙	✓
	Keyphrases	❙	❙	❙	✓	❙	❙	✓	❙	✓	❙	✓	❙	✓	✓	✓	✓	✓
	Regular Expression	❙	❙	✓	❙	❙	❙	❙	❙	❙	❙	❙	❙	❙	❙	❙	✓	❙
Novelty		❙	❙	❙	❙	❙	❙	❙	❙	❙	✓	❙	❙	❙	❙	❙	❙	❙
Data Enrichment	From External Resources	❙	✓	✓	❙	✓	✓	✓	❙	❙	✓	✓	✓	❙	❙	✓	❙	✓
	From Internal Resources	✓	✓	✓	❙	❙	❙	❙	❙	❙	✓	✓	❙	❙	❙	❙	❙	❙

**Table 6:** Exploratory Search Applications Summary. All links have been last accessed in 4 April 2022.

System		Vidar-19 [60]	TopicMaps [58]	Network Visualisations [35]	SciSight [59]	Semviz [43]	EvidenceMiner [56]
Available Charts	Pie Chart	❙	❙	❙	❙	❙	✓
	Histogram	✓	✓	❙	✓	❙	❙
	Data Tables	❙	✓	❙	❙	✓	❙
	Heat Map	❙	❙	❙	❙	✓	❙
	Tile Chart	✓	❙	❙	❙	❙	❙
	Word Cloud	❙	✓	❙	✓	✓	❙
	Stacked Barplot	✓	❙	❙	❙	❙	❙
	Bar Plot	❙	❙	❙	❙	✓	✓
	Bubble Maps	❙	✓	❙	❙	❙	❙
	Network/Graph	❙	❙	✓	✓	❙	❙
	Chord Diagram	❙	❙	❙	✓	❙	❙
Indicators	Frequency	✓	✓	✓	✓	❙	❙
	Count	✓	✓	✓	✓	✓	✓
	Other Indicators	❙	✓	❙	❙	❙	❙
Related Tasks	IE	✓	✓	✓	✓	✓	✓
	Topic Modeling	❙	✓	❙	✓	❙	❙
	NER	❙	❙	✓	✓	✓	❙
	Network Analysis	❙	❙	✓	✓	❙	❙
Data Source	Raw Text	✓	✓	❙	✓	✓	✓
	KG	❙	❙	✓	✓	✓	✓
Reactivity		✓	✓	✓	✓	✓	✓
Public Availability		✓ https://fran6wol.eu.pythonanywhere.com/	✓ http://strategicfutures.org/TopicMaps/COVID-19/dimensions.html	✓ https://nlp.inspirata.com/NetworkVisualisations/TitleNetwork/, https://nlp.inspirata.com/NetworkVisualisations/TreatmentNetwork/, https://nlp.inspirata.com/NetworkVisualisations/LungNetwork/, https://nlp.inspirata.com/NetworkVisualisations/CardioNetwork/	✓ https://scisight.apps.allenai.org/	✓ https://www.semviz.org/	✓ https://evidenceminer.firebaseapp.com/analytics?kw=CORONAVIRUS&corpus=COVID-19

## Data Availability

The data presented in this study are available in the article.
